# Design and methods for a randomized clinical trial comparing three outreach efforts to improve screening mammography adherence

**DOI:** 10.1186/1472-6963-11-145

**Published:** 2011-06-03

**Authors:** Mary E Costanza, Roger Luckmann, Mary Jo White, Milagros C Rosal, Caroline Cranos, George Reed, Robin Clark, Susan Sama, Robert Yood

**Affiliations:** 1 Department of Medicine, University of Massachusetts Medical School, 55 Lake Avenue North, Worcester, MA 01655, USA; 2 Department of Family Medicine and Community Health, University of Massachusetts Medical School, 55 Lake Avenue North, Worcester, MA 01655, USA; 3 Center for Health Policy and Research, University of Massachusetts Medical School, 55 Lake Avenue North, Worcester, MA 01655, USA; 4 Fallon Clinic, 640 Lincoln Street, Worcester, MA 01605, USA

## Abstract

**Background:**

Despite the demonstrated need to increase screening mammography utilization and strong evidence that mail and telephone outreach to women can increase screening, most managed care organizations have not adopted comprehensive outreach programs. The uncertainty about optimum strategies and cost effectiveness have retarded widespread acceptance. While 70% of women report getting a mammogram within the prior 2 years, repeat mammography rates are less than 50%. This 5-year study is conducted though a Central Massachusetts healthcare plan and affiliated clinic. All womenhave adequate health insurance to cover the test.

**Methods/Design:**

This randomized study compares 3 arms: reminder letter alone; reminder letter plus reminder call; reminder letter plus a second reminder and booklet plus a counselor call. All calls provide women with the opportunity to schedule a mammogram in a reasonable time. The invention period will span 4 years and include repeat attempts. The counselor arm is designed to educate, motivate and counsel women in an effort to alleviate PCP burden.

All women who have been in the healthcare plan for 24 months and who have a current primary care provider (PCP) and who are aged 51-84 are randomized to 1 of 3 arms. Interventions are limited to women who become ≥18 months from a prior mammogram. Women and their physicians may opt out of the intervention study.

Measurement of completed mammograms will use plan billing records and clinic electronic records. The primary outcome is the proportion of women continuously enrolled for ≥24 months who have had ≥1 mammogram in the last 24 months. Secondary outcomes include the number of women who need repeat interventions. The cost effectiveness analysis will measure all costs from the provider perspective.

**Discussion:**

So far, 18,509 women aged 51-84 have been enrolled into our tracking database and were randomized into one of three arms. At baseline, 5,223 women were eligible for an intervention. We anticipate that the outcome will provide firm data about the maximal effectiveness as well as the cost effectiveness of the interventions both for increasing the mammography rate and the repeat mammography rate.

**Trial registration:**

http://clinicaltrials.gov/NCT01332032

## Background

Despite the demonstrated need to increase screening mammography utilization and the strong evidence that mail and telephone outreach to women can increase screening in the short term, most managed care organizations have not adopted comprehensive outreach programs for several reasons: 1) the continuing uncertainty about optimum outreach strategy, 2) the absence of practical and replicable models of such systems and 3) the lack of cost effectiveness data to guide decision-making. We undertook this study to compare three successful methods of promoting regular screening mammography (mailed reminders, reminder calls, and more intensive counseling calls) and to identify the most effective and most cost effective of these outreach strategies. Our findings should provide much needed and timely guidance for health plans [1] and Accountable Care Organizations (ACOs).

Determining the most effective way to reach women remains an urgent yet unfinished task. Although public health efforts have been promoting mammography for 25 years or more, national screening rates appear to have stalled. While 81% of American women, 50-74, report having a mammogram in the previous two years [2], only 46% woman get them *regularly *every 1-2 years [3]. A suboptimal level of screening is of considerable concern because the reduction in breast cancer mortality seen with screening mammography requires repeat mammography every several years [4,5]. Non-adherent women are at increased risk for developing advanced or non-curable breast cancers when compared to adherent women [6].

The three arms of the study are:

1) RL: A mailed reminder to schedule a mammogram. Mailed reminders have been shown to increase screening mammography rates by 25-50% [7-11] and have become usual care in some managed care organizations.

2) RC: A mailed reminder followed, if no response, by a reminder telephone call with facilitated access to mammography scheduling. Reminder calls have generally been more effective than reminder letters, especially when the former include the opportunity to schedule a mammogram [12,13]. It is important to confirm this finding in a large population of non-adherent women and especially in women long overdue for a mammogram.

3). ETTC: A mailed reminder followed, if no response, by a mailed educational booklet and, if still no response, by an enhanced tailored telephone counseling call (ETTC). Standard tailored telephone counseling (TTC) has been effective in a wide variety of health promotion outreach efforts [14-21] including efforts focused on cancer prevention and screening [22-25], breast cancer prevention [26] and breast cancer screening [27-33]. TTC has been most effective in breast cancer screening studies when it is used with women who have had a prior mammogram or a recent mammogram but less so in women who had never had a mammogram [27]. Overall, TTC interventions show an improvement in mammography completion rates over mailed reminders or mailed educational materials [13,34-37]. While TTC is often confined to tailored barriers [38], reluctant women may need more encouragement. We developed an enhanced TTC (ETTC) by adding techniques from motivational interviewing [39-43], informative print material and the ability to schedule mammogram on the call. We have pilot tested ETTC and it is effective in helping long overdue women schedule and complete screening mammograms [44,45].

### Study goals

The study goals are:

1) To compare the effectiveness of the three outreach interventions in increasing adherence to mammography screening guidelines in a large managed care population.

2) To identify ways to improve the efficiency and sequencing of the interventions by identifying patient factors and intervention mechanisms associated with increased intervention effectiveness through the evaluation of intermediate outcomes and sub-group analyses.

3) To determine the incremental cost-effectiveness of each telephone intervention compared to the reminder letter and of enhanced tailored telephone counseling to a reminder/scheduling call.

## Methods/design

### Study Design

#### Overall design

**Figure 1:**
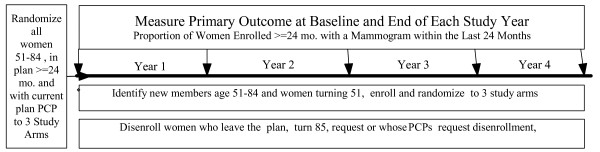
**Overview of study design**.

Figure 1 shows the overall design of the study. Beginning in July, 2009, all eligible women are automatically enrolled and randomized 1:1:1 to the three study arms. After approval by PCPs and passive consent, women are entered into the reminder study. The interventions (letters and calls) are directed only to women who have not had a mammogram in ≥ 18 months. The primary outcome is the proportion of all women who have been enrolled for ≥ 24 months with a mammogram within the previous 24 months. This outcome will be determined at five times, at baseline and at the end of each intervention year. Outcome at the end of the last intervention year will be the single most important result.

### Participants

All women aged 51-84 in the Fallon Community Health Plan (FCHP), for at least 24 months and have a primary care practitioner (PCP) at Fallon Clinic (FC) are eligible and are randomized to one of the three study arms. Exceptions are women with bilateral mastectomies, in hospice, in permanent nursing home care or those mentally incompetent. There are approximately 20,000 such eligible women ages enrolled in the plan at any given time. As women join the health plan or become 51, once in the plan for 24 months, they are enrolled and randomized into the study cohort. Enrollment into the reminder intervention study requires Primary Care Physician (PCP) approval and passive consent of the woman. PCPs are asked to exclude women using the exclusion criteria (See Table 1). Participants are given an opportunity to opt out of the study prior to being intervened, at the time of any intervention and during any phone call.

**Table 1:** Eligibility Criteria

Inclusion Criteria	Exclusion Criteria
1. Age 51-84	1. History of bilateral mastectomies
2. Current patient of Fallon Clinic PCP	2. Significant cognitive impairment
3. Currently enrolled in FCHP	3. Serious illness, precluding screening
4. Has a working telephone	4. Life expectancy <5 years

### Study Setting

#### Study team

The team includes University of Massachusetts researchers (the study joint principal investigators, project manager, psychologist, health economist, biostatistician) and the Fallon Clinic researchers (the site manager, physician liaison, data analyst, schedulers and counselors). The study was approved by the Institutional Review Board for the Fallon Clinic/Fallon Community Health Plan Research Review Committee (IRB # 1191). The study will be conducted in compliance with the Helsinki Declaration.

#### Fallon Community Health Plan (FCHP) and Fallon Clinic Inc. (FC)

FC is a multi-specialty medical group located in Central Massachusetts with about 250 physicians and 20 medical centers. FC is the main group practice affiliated with FCHP, a mixed-model health maintenance organization (HMO) primarily serving the Central Massachusetts area. Automated tables in the FCHP "Data Warehouse" include in- and outpatient visits, laboratory tests and results, radiology procedures and membership. The FC maintains a centralized data repository with automatic feeds into the electronic record. Because most FC women get their mammograms at FC radiology sites, mammogram scheduled and completed dates are readily accessible. Completion data from non-FC sites are captured in billing and medical records. Although racial characteristics of FCHP members are not systematically captured, they reflect that of the Worcester Metropolitan area, which is about 90% White and 10% non-White.

#### Health Plan reminder activities include

An automated reminder call to women a few days before a scheduled mammogram appointment; an annual report to PCPs with the names of women failing to keep their mammogram appointments; at outpatient visits, PCPs receive a reminder in the electronic health record about overdue routine tests, including mammography status. PCPs are neither rewarded nor penalized for their responses.

#### Provider Responsibilities

109 of 111 FC PCPs agreed to: 1) participate in the study and 2) allow their patients to join the study, 3) review the list of their potentially eligible patients, 4) eliminate those having an exclusion criterion and 5) approve their electronic signature for personalized reminder letters. Two PCPs declined steps 3-5 and those functions were carried out by the FC study clinician.

### Interventions

**Figure 2:**
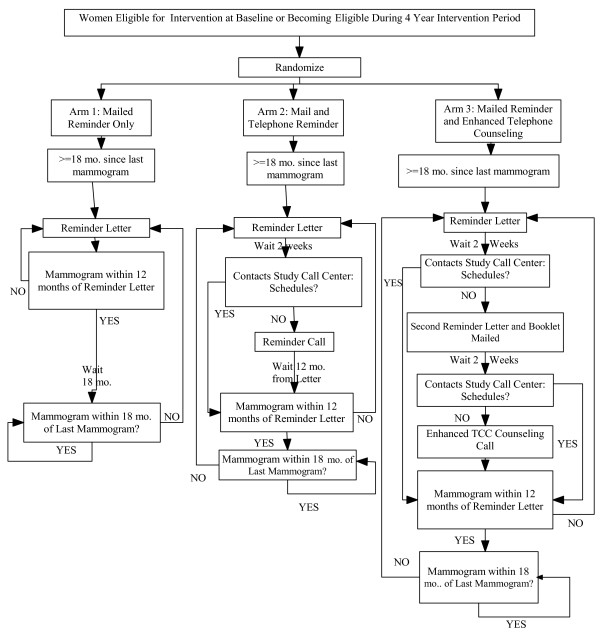
**Overview of Intervention Protocol Implementation**.

Only women ≥ 18 months from a mammogram receive study interventions. See Figure 2. When women are ≥ 18 months from their last mammogram, they all receive the same reminder letter. The letter is electronically signed by a woman's PCP and states: 1) there is no record of a mammogram in the last 18 months, 2) getting regular mammograms every 1-2 years is important and 3) her PCP recommends that she call the Study Scheduler for a mammogram appointment. When women call, the Study Scheduler uses the same script for all, except as noted below for women in the counseling arm. (See Table 2 for Study Scheduler Protocol)

**Table 2:** Study Scheduler Script for Incoming Calls

1. Confirm eligibility.
2. Obtain verbal consent for the baseline survey.
3. Stage the subject if the subject is unsure about scheduling, using Tables 3 and 4.
4. Schedule the mammogram. If the appointment is <= 24 months from the prior mammogram, reaffirm the importance of keeping on a 12-24 month interval schedule, and remind her to keep the appointment.
5. If a woman wants to schedule or confirm an appointment that is inappropriately far in the future, deliver a brief message reminding the woman that Fallon PCPs recommend mammograms every 12-24 months as the best way to find curable breast cancers. Offer to schedule at a more appropriate interval.
6. Administer the brief baseline survey questions that has 6 socio-demographic questions about ethnicity/race, educational level, working status, smoking, marital and income status.

#### RL Arm (reminder letter)

The reminder letter is the only intervention for women in this arm

#### RC Arm (reminder letter followed by Study Scheduler call)

All women receive a reminder letter as above. If a woman *does not call *following receipt of the reminder letter or does not schedule a mammogram on her own within two weeks of the reminder letter, the Study Scheduler makes up to five call-attempts to reach her. The script is the same as noted above.

#### ETTC Arm: (reminder letters and booklet followed by Counselor call)

All women receive a reminder letter as above. If a woman calls following receipt of the reminder letter, the Study Scheduler uses the same script as in Table 2 but replaces item #4 with "Inform the woman that she will receive a mammography booklet but will not get a Counselor call unless she would like to". If a woman calls in to schedule or confirm a mammogram appointment that is inappropriately far in the future, the Scheduler sends a mammography booklet and makes an appointment for her to receive a Counselor call.

If after receiving the reminder letter, a woman schedules a mammogram on her own, there is no further intervention. If a woman in the counseling arm does not call within two weeks after the first reminder letter, a mammography booklet is sent with a second reminder letter that states that if she does not call or schedule a mammogram within two weeks, a Counselor will call her. If the woman does not respond to the second reminder letter, the Counselor will make five call-attempts.

Counseling calls typically last about 30-35 minutes. If a subject requires a repeat counseling call during the study period, the call content will be different from that of the initial call. During repeat calls, the Counselor will address any change in the subject's stage of readiness, beliefs and barriers. The Counselor protocol using the CATI (computer assisted telephone interviewing) system was detailed in our previous publication [44,45].

Theoretical Basis of the Counseling Intervention:

1. Our counseling script incorporates a stage-based theory of behavior change that emphasizes the evolution of knowledge and personal risk perception in the development of an intention to act, and the variables that then influence acting on that intention [46,47]. The stage-based models have attempted to integrate the conceptual bases and empirical findings of the older models of change to capture the complexities of health behavior. The Precaution Adoption Process Model (PAPM) [48,49] is useful in working with non-adherent women because it classifies them into four stages that provide a more detailed description of the earlier stages of decision-making. In designing the ETTC counseling algorithm, we used our pilot mammography study to adapt the PAPM stages (See Tables 3 &4) [44,45].

**Table 3:** Staging A Woman's Readiness to Get A Mammogram

STAGE	PAPM STAGES REVISED
1	Unaware - of mammography and guidelines

2	Unengaged - aware but does not see need to get mammogram

3	Undecided- considering the pros and cons of mammograms

4A	Definite No - would never get a mammogram

4B	Qualified No - but might reconsider

5A	Definite Yes - planning sometime

5B	Definite Yes - planning soon but cannot set date

5C	Definite Yes - planning soon and can set date

**Table 4:** Initial Staging Questions

• For those with no record of a mammogram: "Have you ever had a mammogram"?
• If never had a mammogram: "Have you ever heard about a mammogram" "Do you know what a mammogram is?"
• For those who have had a mammogram: "Are you planning or thinking about getting a mammogram?"
• If planning: "Within 3 months?" "More than 3 months?" " Don't know when"
• If planning: "Can I schedule you now? When?"
• If not planning: "Have you decided not to get a mammogram?"
• If decided no: "Have you thought seriously about whether mammograms are for you?"
"Are you unable to decide to get a mammogram?" "Do you mean you would never get a mammogram or you would not get a mammogram unless something happened?"

2. The ETTC counseling algorithm interviewing and script use the following (MI) techniques [39]: 1) expressing empathy through reflective listening skills and a warm empathetic style; 2) developing discrepancy between the patient's current decision and future goals so that the patient verbalizes the need for screening; 3) avoiding argumentation; 4) "rolling with" resistance instead of arguing with it; 5) involving the patient in active problem solving; 6) supporting self-efficacy for screening change by facilitating the patient's own action plan for mammography completion.

#### Counselor and Study Scheduler Recruitment, Training and Supervision

The telephone counselors are women with Master's level education or counseling experience. The Counselors train for two weeks in the detailed counseling protocol by a clinical psychologist and an experienced telephone counselor. Training includes: building adequate skills using the CATI system, counseling protocol and materials; role-playing all aspects of the intervention from staging patients to action planning and familiarizing the Counselor with the scheduling procedures. Study Schedulers are Bachelor's prepared Research Assistants with some research experience. The initial training for the Study Scheduler takes 20 hours. Both Counselors and Study Schedulers received didactic presentations on research methods, study goals, breast cancer and mammography screening, systems-based interventions to increase mammography use, screening rates in minority populations and role-playing with study staff and "mock" patients. Selected telephone calls are audio taped and reviewed by the study project manager for quality control and to insure fidelity to protocol. Appropriate refresher/corrective sessions are undertaken as needed.

#### Repeat interventions (booster doses)

There are very few studies examining the effect of repeat interventions [13,50-53]. Since the intervention period will span 48 months, we will be able to evaluate the booster effect more fully.

### Outcomes

The primary outcome is the proportion of women continuously enrolled for ≥24 months who have had ≥1 mammogram in the last 24 months. This measure mimics the standard HEDIS (Healthcare Effectiveness and Data Information Set) measure used by most health plans and systems to track mammography utilization, although the HEDIS measure is usually applied only to women age 50-69. The measure is a cross-sectional, prevalence measure that can be applied in a series of static "snapshots" to a dynamically changing population like the one in our study and in any typical health plan.

The secondary outcomes are 1) the number of women in each arm requiring repeat interventions and 2) the immediate outcomes (change in stage of readiness, mammography scheduling for those in the RC and ETTC arms during calls) and short term outcomes (receiving a mammogram within 3 months) following the interventions, for all women receiving the interventions for the first time, adjusted as needed by appropriate covariates (e.g. age, time to last mammogram, marital status and other factors).

The main cost-effectiveness outcome is the incremental cost-effectiveness of each intervention combination, that is, the difference in the percentage of eligible women with mammograms divided by the difference in total cost for each intervention pair. The study also measures separately the startup and operating costs of each intervention.

### Data Collection and Management

During the four-year intervention period the claims database and the electronic health record data base are searched weekly to determine eligibility of women to receive a reminder letter. Study staff enter data on study enrollment when they receive information from PCPs and/or from women relevant to enrollment. The only data required for primary outcome measures on women enrolled in the study are duration of enrollment in FCHP/FC, dates of mammograms, and birth date for developing age-specific outcomes.

Secondary analyses will include subgroup analyses involving the primary outcome, analyses involving secondary outcomes (e.g. change in stage of readiness from beginning to the end of reminder and ETTC calls, completion of scheduled mammograms and time from intervention to mammogram completion) and other analyses requiring independent variables collected from the FCHP database and during all calls from and to subjects. Medicare and Medicaid insurance coverage, history of claims for preventive checkups and other screening tests (e.g. Pap smears, cholesterol, colon cancer screening) will be obtained from FCHP claims databases and the electronic health record database to compare adherence to mammography recommendations to the utilization of other preventive services. (Table 5 shows all of the data sources)

**Table 5:** Data Sources for Analysis

Data Sources	Primary Outcome Analysis	Secondary Data Analyses	Process Analysis
FCHP and FC Membership Database	X	X	X

FCHP Claims Database and Electronic Health Record database	X	X	X

Fallon Clinic Scheduling System		X	X

Patient Survey (Administered to subjects calling the Study Scheduler or receiving reminder or ETTC calls)		X	

ETTC and Reminder Call CATI System & Tracking Database		X	X

Phone Call Logs			X

PCP Approvals for Patient Enrollment	X	X	X

Patient Refusals of Enrollment	X	X	

The cost effectiveness analysis will measure all costs from the provider perspective. We will estimate start-up costs and fully staffed operational and cost estimates to give potential adopters a sense of the investment required to implement and maintain each intervention. Start up costs include database and software development and installation, equipment purchases, training costs, and overall administrative costs for the RC and ETTC interventions. Operational costs include staff and administration salaries, overhead costs (office space, phone, electric, etc.), office supplies, information system and equipment maintenance, and postage. We employ a "micro-costing" approach where units for each component of resource use are measured directly, a unit cost is applied for each and units are multiplied by unit cost to get cost estimates. Staff time and effort involved for all tasks in the intervention are measured using staff logs.

### Sample Size and Power

#### Power and Sample Size

Given, the typical size of the female population ages 51-84 at FCHP/FC of 25,000, we estimated there would be at minimum 15,000 women available for the analyses involving the primary outcome at each year. We explored power to detect differences in primary outcome among intervention groups under two scenarios. Based on findings from our EPICS study [27] we believe that it is reasonable to expect differences of 3-5% or greater across groups. Differences that are smaller would likely be of little interest to health plans. In the first scenario we assumed that the primary outcome in the RL group remains at 75%, as it is now and that the outcome in the RC group is 80% and in the ETTC 85%. In the second scenario we assumed these outcomes: RL 80%, RC 83% and ETTC 86%. With 15,000 in the sample (5,000 per group) there is greater than 99% power to detect the differences proposed or larger ones across groups under both scenarios. For the trends over time there will be approximately 90% power for an increasing trend over five years leading to a 5% increase in any group (e.g..75 to.80) based on Cochran-Armitage test of trend with power calculations described by Hintze [54]. Power calculations were carried out using Power Analysis and Sample Size (PASS) 2008.

#### Proportion needing repeat interventions

The comparison of rates of repeat interventions will be made between the two call arms of the study (RC and ETTC). We identified sufficient power (greater than 85%) for differences in proportion needing repeat intervention of 6% (80% vs. 86% for example).

### Statistical Methods

#### Primary Outcome Analysis: HEDIS-like measure over time

The unit of randomization and analysis is the individual woman. The primary outcome measure is the proportion of women continuously enrolled for 24 months with ≥1 mammogram in the past 24 months. This will be evaluated at the five measurement time points. The main study finding of interest will be the comparison of this proportion across groups after the last year of intervention (Year 4), because we believe this result best illustrates the magnitude of intervention effect that would be achieved in all future years. The second hypothesis is that the primary outcome measure may change differently over time in the telephone intervention groups because there may be some cumulative effect of repeated interventions on women who do not respond the first time. It is also possible that the interventions may become less effective when applied repeatedly to the same group of women over time. Changes in the composition of the patient population over time could also cause changes in the outcomes. We will estimate the trend over time of the primary outcome within each intervention group and compare the trends over time among the randomized groups. The third hypothesis is that lower age will be associated with a greater response to less intensive interventions. We will investigate this hypothesis through analyses assessing the interaction of subgroups and the intervention arms. To accomplish this and some related analyses involving the primary outcome, we will examine the interactions with intervention group of age, type of medical insurance, and time since last mammogram. Analyses will be carried out using generalized linear models. More specifically, logistic regression models will provide estimates of associations of factors (including the intervention groups) with the primary outcome of mammography screening and adjustment for possible confounders. For models over time with the panel data we will appropriately adjust for correlations using robust variance estimation.

#### Secondary Outcomes Analyses

We hypothesize that fewer women who receive the intensive telephone intervention will require a repeat telephone intervention in subsequent years compared to women receiving the brief intervention. For this analysis we will assemble two cohorts of women, one that received ETTC and subsequently received a mammogram within 12 months of the call and another that received a reminder call (RC) and also received a mammogram within 12 months of the call. Proportions scheduling a mammogram within 18 months of the index mammogram and completing a mammogram within 19 months of the index mammogram will be compared between the two intervention arms.

The second hypothesis is that more women who schedule a mammogram after intensive counseling than after a brief reminder call will complete the mammogram. Other secondary analyses will include: 1) analysis of immediate term outcomes (change in stage of readiness, mammography scheduling) and short term outcomes (receiving a mammogram within three months) following telephone interventions, for all women receiving the interventions for the first time, adjusted as needed by appropriate covariates (e.g. age, time to last mammogram, marital status and other factors), 2) response to reminder letters measured by the proportion of women without a recent mammogram scheduled or completed who schedule a mammogram within two weeks of a reminder letter, compared to the proportion of women scheduling during other two week intervals, adjusted for age, time to last mammogram, and other factors as appropriate.

#### Cost and Cost-Effectiveness Analyses (CEA)

Following similar studies of methods to increase mammography screening, we will conduct the analysis from the perspective of the payer/provider, FCHP/FC [55,56], rather than from the perspective society typically advocated by cost-effectiveness guidelines. Our rationale is that provider costs are the most relevant measure for groups considering adopting the study interventions. Separate comparisons will be conducted for each intervention year including calculating total startup costs for the ETTC and the RC group; calculating the cost of operating each intervention by summing costs for each intervention in each of the four intervention years. Costs for ETTC and RC will be calculated by amortizing startup costs across the four-year intervention period and adding the operating costs in each year of the intervention. Costs for RL intervention will be based on operating costs only. Recent cancer screening studies have compared differences in total, rather than average, costs across interventions [55-57]. To estimate the incremental cost-effectiveness of each intervention combination, we will compare the difference in the percentage of eligible women with mammograms divided by the difference in total cost for each intervention pair. We will report initial results as the incremental cost of increasing on-time mammograms by 1%. This figure can also be converted to an estimated cost per additional mammogram for a hypothetical sample of women. We will conduct sensitivity analyses to determine the effect of including or excluding startup costs, excluding administrative overhead costs, amortizing startup costs over a longer or shorter period, higher or lower (e.g. 10%) costs for each intervention and higher or lower effectiveness (e.g. 5% and 10% variation.)

### Randomization

Patients joining the FCHP are automatically entered into our tracking system if they are female, aged 51-84, have a FC primary care provider and are in the plan for 24 months. Once identified and loaded onto the tracking system, they are automatically randomized to one of the three study arms. No blocking or stratification is used. The tracking database server uses a built-in T-SQL function to randomly allocate a number from 1-3 to each patient. The numbers refer to the specific intervention arm assignment. Documentation of the function can be found at: http://msdn.microsoft.com/en-us/library/ms177610.aspx

### Blinding

Since women receive different intervention depending on the arm to which they were randomized, blinding of subjects and the staff that receives and makes calls is not possible. Although a woman might reveal what type of contact she had had with study personnel in discussion with her PCP, PCPs are not informed as to which arm a patient is in. While the intervention assignment and the individual mammography outcome is not blinded, overall study arm outcomes will be blinded for all study personnel while the study is ongoing. The statistical team will conduct outcome analyses only at the conclusion of the intervention phase.

### Baseline results

Since July 2009, 18,509 female FCHP/FC enrollees aged 51-84 have been enrolled into our tracking database and were randomized into one of three arms. Table 6 shows their mammogram status at randomization. Table 7 shows the characteristics of the study sample, their approval or non-consent status by age. At baseline, 5,223 women were eligible for an intervention.

**Table 6:** Mammogram Status at Study Entry*

Last Mammogram	<8 mo. ago	18-23 mo. ago	24-29 mo. ago	30-35 mo. ago	26-48 mo. ago	>48 mo. ago
N = 18,509 (100.0%)	12,808 (69.20)	986 (5.33)	866 (4.68)	599 (3.24)	851 (4.60)	1,758 (9.50)

* Columns do not total to 100% because of missing data

**Table 7:** Characteristics of Study Sample

Age	Eligible women at baseline in study database*	Women opted out of intervention phase after broadcast letter	PCP approved for possible intervention phase	Women opted out of intervention phase at 1^st ^intervention attempt	Women eligible for reminder intervention study	Women >= 18 months from last mammogram and intervention eligible
50-59	5,901 (31.88)	30 (3.44)	5,826 (33.62)	12 (20.69)	5,814 (33.66)	1,842 (35.27)

60-69	4,781 (25.83)	115 (13.17)	4,613 (26.62)	13 (22.41)	4,600 (26.63)	1,146 (21.94)

70-79	5,429 (29.33)	494 (56.59)	4,818 (27.80)	15 (25.86)	4,803 (27.81)	1,329 (25.45)

80-84	2,398 (12.96)	234 (26.80)	2,074 (11.97)	18 (31.03)	2,056 (11.90)	906 (17.35)

Total	18,509	873	17,331	58	17,273	5,223

*All women 50-84 in FCHP for ≥24 months with current FC PCP and no mammogram in last 4 months

## Discussion

The interventions we will evaluate and compare in this study represent system solutions to reminding, scheduling, counseling and motivating women to get regular repeated mammograms. These interventions represent an effort to remove much of the reminder/counseling burden from PCPs and their practice staff. In particular, we are interested in evaluating the effectiveness of a centralized reminder and counseling system as alternative to the primary care providers' (PCPs) role in reminding, educating and/or motivating women to get screening mammography. While many women may be brought to regular on time screening with minimal reminders, women who are significantly overdue for screening or habitually non-compliant need more than reminders. We believe counseling and motivating these women will be required. Unfortunately, providers have not been consistently involved in lengthy education or counseling the more reluctant women due to time constraints, limited patient responsiveness, limited PCP training in counseling and limited reimbursement for providing preventive services [58]. Even when providers undertake counseling, it may be brief, incomplete and/or relatively ineffective due to limited time and training [59-63]. All these problems are compounded by the fact that the numbers of new physicians entering primary care are decreasing significantly and the need for physician extenders is increasing [64]. Finding cost-effective ways to complement PCPs recommendations is critical and the results of this study will be illuminating. In addition, since this study aims to identify the most effective and most cost effective of the outreach strategies, the forthcoming result will provide much needed and timely guidance for health plans [1] and ACOs.

Since the intervention period will span 48 months, we will be able to evaluate the effect of repeated interventions more fully than others have [53]. We hypothesize that woman who respond to an initial intervention may, when coming due for another mammogram, either requires no intervention or a less intense intervention than they responded to initially.

## List of Abbreviations

ACOs: Accountable Care Organizations; CATI: Computer assisted telephone interview; CEA: Cost effectiveness analysis; EPICS: Empowering Physicians to Improve (Breast) Cancer Screening; ETTC: Enhanced tailored telephone counseling call; FC: Fallon Clinic; FCHP: Fallon Community Health Plan; HEDIS: Healthcare Effectiveness and Data Information Set; PAPM: Precaution Adoption Process Model; PASS: Power Analysis and Sample Size; PCP: Primary Care Provider; RC: Reminder call; RL: Reminder letter; TTC: Tailored telephone counseling

## Competing interests

The authors declare that they have no competing interests.

## Authors' contributions

MEC, RL, MJW, MR, SS, RY participated in the design of the study. MEC, RL, MJW, MR, CC, SS participated in the conceptualization of the interventions. SS, RY contributed to the recruitment of subjects. MEC, RL, MJW, MR, CC, SS, RY participated in implementation of the study. MEC, RL, SS, GR, RC participated in the development of outcome measurement. GR is the biostatistician. All authors reviewed and approved of the final manuscript.

## Pre-publication history

The pre-publication history for this paper can be accessed here:

http://www.biomedcentral.com/1472-6963/11/145/prepub
